# The overlooked problem among surgical patients: Preoperative anxiety at Ethiopian University Hospital

**DOI:** 10.3389/fmed.2022.912743

**Published:** 2022-08-02

**Authors:** Yophtahe Woldegerima Berhe, Tadesse Belayneh Melkie, Girmay Fitiwi Lema, Marye Getnet, Wubie Birlie Chekol

**Affiliations:** ^1^Department of Anesthesia, University of Gondar, Gondar, Ethiopia; ^2^Department of Emergency and Critical Care Nursing, University of Gondar, Gondar, Ethiopia

**Keywords:** preoperative anxiety, anxiety, anxiety of anesthesia and surgery, state-trait anxiety inventory, preoperative care

## Abstract

**Introduction:**

Anxiety was repeatedly reported as the worst aspect of the perioperative time. The objective of this study was to assess the prevalence of preoperative anxiety among adult surgical patients at University of Gondar Comprehensive Specialized Hospital (UoGCSH), Northwest Ethiopia.

**Methodology:**

A hospital-based cross-sectional study was conducted among surgical patients at the university hospital. After obtaining ethical approval, 407 surgical patients were approached during the preoperative period. Preoperative anxiety was assessed by State-Trait Anxiety Inventory. The association between variables was determined by using binary logistic regression analysis. Strength of association was described in adjusted odds ratio (AOR), and a *p*-value < 0.05 at 95% confidence interval was considered statistically significant.

**Results:**

A total of 400 patients were included in this study with a 98.3% response rate. Preoperative anxiety was observed among 237 (59.3%) patients, and the median (IQR) STAI score was 50 (40–56.7); age, ≥ 60 years (AOR: 5.7, CI: 1.6–20.4, *P*: 0.007); emergency surgery (AOR: 2.5, CI: 1.3–4.7, *P*: 0.005); preoperative pain (AOR: 2.6, CI: 1.2–5.4, *P*: 0.005); and rural residency (AOR: 1.8, CI: 1.1–2.9, *P*: 0.031) were found significantly associated with preoperative anxiety.

**Conclusion:**

The prevalence of preoperative anxiety among surgical patients was high. Older age (≥ 60 years), emergency surgery, preoperative pain, and rural residency were found significantly associated with preoperative anxiety. Assessment for preoperative anxiety should be a routine component of preoperative assessment of both elective and emergency surgical patients. Preoperative pain should be appropriately managed as it can help to reduce preoperative anxiety. Optimal anxiety reduction methods should be investigated and implemented in the hospital.

## Introduction

Anxiety is defined as emotional response to anticipation of future or imminent threat, which can be real or perceived (1). It is a response to external or internal stimuli that can have behavioral, emotional, cognitive, and physical symptoms (2). The perioperative period is one of the most disturbing situations for most of surgical patients. As a result, anxiety is repeatedly reported as the worst aspect of the preoperative period particularly (2, 3).

Preoperative anxiety is common and affects both anesthetic management and overall surgical outcomes (4). Although some levels of anxiety are expected during the preoperative period, it can become a clinical problem when exaggerated and associated with excessive fear and multi-systemic manifestations (5, 6). Preoperative anxiety is known to increase anesthetic agent requirement, delayed awaking, hemodynamic derangements, postoperative pain, and delay in wound healing, risk of infection, cancelation, hospital stay, and dissatisfaction. All of these have direct and indirect cost implications, especially for hospitals in the low- and middle-income countries (4–12). Furthermore, after cardiac surgery, preoperative anxiety was found to be an independent predictor for long-term morbidity and mortality (11, 13, 14).

The prevalence of preoperative anxiety varies according to the types of surgery, gender, definition used, motives for surgery, and educational status. Evidence has shown that more than a half of surgical patients experience preoperative anxiety (15–19). In Ethiopia, limited studies have been conducted on preoperative anxiety that estimated its prevalence between 47 and 70.3% (15, 20–22).

Despite disputing results, a previous study has documented various factors associated with preoperative anxiety (2, 5, 6, 15–17, 22–33). Increasing age, male/female, having low educational status, living in rural residency, poor financial status, poor social support, history of alcohol/substance use, types and extent of proposed surgery, general anesthesia, previous surgical experiences, personal susceptibility to stressful situations, and American Association of Anesthesiologists (ASA) physical status were found to be associated with preoperative anxiety (2, 5, 6, 15–17, 22–33).

Even though preoperative anxiety has several negative consequences both during and after surgery, it has been given less attention by the clinicians in our hospital and not well studied in our country (22). Therefore, this study aimed to determine the magnitude and factors associated with preoperative anxiety among adult surgical patients in our hospital [University of Gondar Comprehensive Specialized Hospital (UoGCSH)] using the preoperative scale-short form.

## Methodology

A hospital-based, cross-sectional study was conducted at UoGCSH from April 1—May 30, 2020. The hospital is found in Gondar town, Northwest Ethiopia. During the data collection time, the hospital had 4 general, 2 obstetric, and 2 gynecologic operation theaters. All consecutive adult (18 +) patients who came for both elective and emergency surgical operations during the study period were eligible to be included in the study unless they were unwilling to participate, unable to communicate, diagnosed for preexisting anxiety and other psychiatric disorders, and unable to understand Amharic or English languages.

The outcome variable was the preoperative anxiety level, which was assessed using STAI. The independent variables were socioeconomic-demographic factors (age, sex, educational status, residency, occupational status, and marital status); behavioral variables (smoking, alcoholism, and substance abuse); coexisting medical condition, previous exposure to anesthesia and surgery; type, urgency, and grade of the current surgical operation.

To determine the sample size, a single population proportion formula was used. A study done by Woldegerima et al. at UoGCSH, Northwest Ethiopia, reported that the prevalence of preoperative anxiety was 59.6% (22). By assuming 95% of confidence interval with a 5% margin of error, the sample size for the study was 370, and, when 10% of a non-response rate was added, the total sample size became 407. All consecutive and eligible surgical patients were included.

Ethical approval was obtained from the ethical review committee of Department of Anesthesia, University of Gondar. Written informed consent or finger-print consent was obtained from each study participant after full agreement to be enrolled in the study. A consent form was prepared in English language and the local Amharic language and presented to each participant to read. For those who were unable to read, data collectors read the consent information. Confidentiality was ensured by removing identifiers and locking the questionnaires in a secured area. Additionally, when the patients were found experiencing clinically significant preoperative, the data collectors reported for clinicians (Anesthetists, Surgeons, or Nurses) to provide anxiety management.

To date, STAI, which was developed by Spielberger et al., has been the most commonly used tool to assess preoperative anxiety (34). The original STAI has 40 items, 20 in each sub-scale (state and trait). Short versions of the STAI scale were developed and found valid and as effective as the extended version to measure anxiety. The commonly used short version of STAI was developed by Marteau and Bekker, which is composed of 6 items drawn from the extended version, and its validity and reliability were supported. A score of 44 was used as a cut-off value, above which recognized as clinically significant preoperative anxiety (35, 36).

Data were collected by using a pre-tested structured questionnaire by a well-trained anesthetist to prevent assessor bias. A pre-test was conducted on 21 (5%) patients whose data were not included in the main study, and then necessary corrections were made according to the questionnaire for the main study. Elective surgical patients were admitted at least 2 days prior to the date of the surgery to the corresponding wards where data collectors met them after an anesthetic visit at a night before the surgery. Emergency patients were met at the holding area. Brief information regarding assessment tool (STAI) was provided for the study participants. The collected data were cleaned and analyzed by using SPSS version 20 software (IBM Corporation). Descriptive results were presented by frequency, percentage, mean, and standard deviation. Relationships of nominal data were presented in cross-tabulations. The Omnibus test of fit was used to assess model fitness. To identify factors associated with preoperative anxiety, bivariate binary logistic regression analyses were performed and followed by multivariate binary logistic regression analyses. Variables with *p*-value < 0.2 in the bivariate analyses were fitted to the final multivariate analyses. Results were presented in the crude and adjusted odds ratio. Variables with a *p*-value < 0.05 in the final multivariate analysis were considered to have statistically significant association at a 95% confidence interval (CI).

## Results

A total of 407 patients were approached, and data from 400 (98.3%) participants were used for final analysis. Data from 7 (1.7%) patients were excluded due to errors and incompleteness. The 226 (56.7%) patients were female, and majority of the participants –189 (47.3%)—had age between 18 and 29. The median (IQR) age was 30 (25–40) years. The patients who came from rural residencies counted 237 (59.3%). The 168 (42%) participants were illiterate, and only 70 (17.5%) had completed college education. The majority of the participants –300 (75%)—were married (Table 1).

**TABLE 1:** Socio-demographic characteristics of surgical patients, *N* = 400.

Variables	Frequency (n)	Percentage (%)
**Sex**		
Male	174	43.5
Female	226	56.5
**Age**		
18–29	189	47.3
30–39	110	27.5
40–49	52	13.0
50–59	25	6.3
60 and above	24	6.0
**Residency**		
Urban	163	40.8
Rural	237	59.3
**Educational status**		
Illiterate	168	42.0
Primary school	83	20.8
Secondary school	79	19.8
College and above	70	17.5
**Marital status**		
Unmarried	62	15.5
Married	300	75.0
Separated	38	9.5

Regarding clinical characteristics of the surgical patients, slightly more than half of them were classified as ASA 1 and the remaining 195 (48.8%) patients as ASA II. The 148 (37%) patients had previous hospital admissions, while 91 (22.8%) had previous anesthetic exposure. Nearly 2/3rd of the patients came for emergency surgery, and 213 (53.3%) patients had complained for moderate to severe preoperative pain on visual analog scale (VAS). The median (IQR) of preoperative pain was 35 (13–59). Obstetric and general surgical procedures were the leading procedures performed in the hospital [125 (31.3%) and 124 (31%), respectively]. The primary plan of anesthesia was regional anesthesia, 235 (58.8%), and majority of the patients –361 (90.3%)—had reported that they had good patient to anesthetist communication (Table 2).

**TABLE 2:** Health status and clinical characteristics of surgical patients, *N* = 400.

Variables	Frequency (n)	Percentage (%)
**ASA classification**		
I	205	51.3
II	195	48.8
**Previous hospital admission**		
Yes	148	37.0
No	252	63.0
**Previous anesthetic exposure**		
Yes	91	22.8
No	309	77.3
**Severity of preoperative pain**		
No pain	58	14.5
Mild pain	129	32.3
Moderate to severe pain	213	53.3
**Urgency of surgery**		
Elective	145	36.3
Emergency	255	63.8
**Class of surgery**		
General and urologic	124	31.0
Orthopedic	88	22.0
Gynecologic	63	15.8
Obstetric	125	31.3
**Grade of surgery**		
Minor	103	25.8
Intermediate	182	45.5
Major	115	28.8
**Type of planned anesthetic technique**		
General anesthesia	165	41.3
Regional anesthesia	235	58.8
**Patient to anesthetist communication**		
Good	361	90.3
Poor	39	9.8

ASA, American Society of Anesthesiologists.

Preoperative anxiety was observed among 237 (59.3%) patients, and the median (IQR) STAI score was 50 (40–56.7). Cross-tabulations have shown that 66.1% of the male patients and 54% of the female patients have developed clinically significant preoperative anxiety. The patients who had no previous hospital admission and anesthetic exposure were found frequently anxious during the preoperative period than those who had. We noticed preoperative anxiety more frequently (71%) among the patients who came for general and urologic surgical procedures, and lower frequency of preoperative anxiety was observed among the obstetric patients (48.8%). The patients were less frequently anxious when the plan of anesthetic technique was regional anesthesia than general anesthesia (51.9 vs. 69.7%, respectively). The patients who reported poor patient to anesthetist communication were more anxious than those who reported good patient to anesthetist communication (69.2 vs. 58.2%) (Table 3).

**TABLE 3:** Binary logistic regression; factors associated with preoperative anxiety among surgical patients, *N* = 400.

Variables	Anxiety status		OR (95% CI)		*P*-value

	**Non (%)**	**Yes n (%)**	**Crude**	**Adjust**	
**Sex**					
Male	59 (33.9)	115 (66.1)	1.7 (1.1, 2.5)	0.8 (0.3, 1.8)	0.52
Female	104 (46.0)	122 (54.0)	1	1	
**Age**					
18–29	77 (40.7)	112 (59.3)	1	1	
30–39	50 (45.5)	60 (54.5)	0.8 (0.5, 1.3)	1.2 (0.7, 2.0)	
40–49	21 (40.4)	31 (59.6)	1.0 (0.5, 1.9)	1.5 (0.7, 3.2)	
50–59	11 (44.0)	14 (56.0)	0.9 (0.4, 2.0)	1.2 (0.4, 3.3)	
60 and above	4 (16.7)	20 (83.3)	3.4 (1.1, 10.5)	5.7 (1.6, 20.4)	**0.007**
**Educational status**					
Illiterate	67 (39.9)	101 (60.1)	1.2 (0.7, 2.1)	a	
Primary school	32 (38.6)	51 (61.4)	1.3 (0.7, 2.4)		
Secondary school	33 (41.8)	46 (58.2)	1.1 (0.6, 2.1)		
College and above	31 (44.3)	39 (55.7)	1		
**Residency**					
Urban	79 (48.5)	84 (51.5)	1	1	
Rural	84 (35.4)	153 (64.6)	1.7 (1.1, 2.6)	1.8 (1.1, 2.9)	**0.031**
**Previous hospital admission**					
Yes	66 (44.6)	82 (55.4)	1	a	
No	97 (38.5)	155 (61.5)	1.3 (0.9, 1.9)		
**Previous anesthetic exposure**					
Yes	48 (52.7)	43 (47.3)	1	1	
No	115 (37.2)	194 (62.8)	1.9 (1.2, 3.0)	1.3 (0.8, 2.4)	0.32
**Preoperative pain**					
No pain	36 (62.1)	22 (37.9)	1	1	
Mild pain	66 (51.2)	63 (48.8)	1.6 (0.8, 2.9)	1.1 (0.5, 2.4)	
Moderate to severe pain	61 (28.6)	152 (71.4)	(2.2, 7.5)	2.6 (1.2, 5.4)	**0.005**
**Grade of surgery**					
Minor	33 (32.0)	70 (68.0)	1.4 (0.8, 2.4)	1.6 (0.8, 3.3)	0.21
Intermediate	85 (46.7)	97 (53.3)	0.7 (0.5, 1.2)	2.1 (0.9, 4.9)	
Major	45 (39.1)	70 (60.9)	1	1	
**Class of surgery**					
General and urologic	36 (29.0)	88 (71.0)	2.6 (1.5, 4.3)	3.0 (0.9, 10.0)	0.32
Orthopedic	33 (37.5)	55 (62.5)	1.7 (1.0, 3.0)	2.8 (0.9, 9.0)	
Gynecologic	30 (47.6)	33 (52.4)	1.2 (0.6, 2.1)	2.1 (0.6, 6.7)	
Obstetric	64 (51.2)	61 (48.8)	1	1	
**Type of surgery**					
Elective	81 (55.9)	64 (44.1)	1	1	
Emergency	82 (32.2)	173 (67.8)	2.7 (1.8, 4.1)	2.5 (1.3, 4.7)	**0.005**
**Type of planned anesthetic technique**					
General anesthesia	50 (30.3)	115 (69.7)	2.1 (1.4, 3.2)	1.3 (0.6, 2.5)	0.48
Regional anesthesia	113 (48.1)	122 (51.9)	1	1	
**ASA class**					
I	77 (37.6)	128 (62.4)	1.3 (0.9, 2.0)	a	
II	86 (44.1)	109 (55.9)	1		
**Patient to anesthetist communication**					
Good	151 (41.8)	210 (58.2)	1	a	
Poor	12 (30.8)	27 (69.2)	1.6 (0.8, 3.3)		

^a^Not significant in bivariate analysis and failed to appear into the final multivariate analysis.

ASA, American Society of Anesthesiologists.

Bold values are statistically significant in multivariate binary logistic regression analysis.

The final multivariate logistic regression analysis revealed that age ≥ 60 years, rural residency, moderate to severe preoperative pain, and emergency surgery were significantly associated with preoperative anxiety. The patients whose age was 60 and above were found nearly 6 times anxious compared to those whose age was between 18 and 29 (AOR: 5.7, CI: 1.6–20.4, *P*: 0.007). The patients that came from rural residencies had nearly doubled odds to develop preoperative anxiety compared to those who live in urban areas (AOR: 1.8, CI: 1.1–2.9, *P*: 0.031). Presence of moderate to severe pain during the preoperative period increases the occurrence of preoperative anxiety by nearly threefold (AOR: 2.6, CI: 1.2–5.4, *P*: 0.005). The patients who came for emergency surgery were 2.5 times more likely to have preoperative anxiety than those who came for elective surgery (AOR: 2.5, CI: 1.3–4.7, *P*: 0005) (Table 3). Among the emergency surgical patients, most of the patients (71%) had suffered from moderate to severe pain, which, in turn, had significant association with preoperative anxiety (Table 4).

**TABLE 4:** Preoperative pain among surgical patients, *N* = 400.

Variable	No pain	Mild pain	Moderate to severe pain
Elective surgical patients	43 (29.7%)	70 (48.3%)	32 (22.1%)
Emergency surgical patients	15 (5.9%)	59 (23.1%)	181 (71.0%)
Preoperative pain: VAS [median (IQR)]	35 (13–59)		

VAS, Visual Analog Scale; IQR, Inter-quartile Range.

## Discussion

Waiting to undergo surgery is expected to cause terrible feelings that can result in preoperative anxiety (17). When this feeling becomes intensified, it can cause varieties of deleterious impacts on anesthesia and surgery (4). Our study revealed that preoperative anxiety occurred among 237 (59.3%) patients, and previous studies have consistent results to ours (4, 22). The previously reported prevalence of preoperative anxiety has been varied among different patient groups, and according to definitions and measurements utilized to assess (18, 32).

The patients whose age was 60 and above were found nearly 6 times anxious compared to the patients whose age was between 18 and 29 years (AOR: 5.7, CI: 1.6–20.4, *P*: 0.007). Similarly, previous studies have reported high prevalence of preoperative anxiety in elderly patients (37, 38). This association might be explained by comorbidities and frailty that could compromised the elderly’s physiologic reserves to cope with anesthesia and surgery (39, 40). In contrast, multiple studies have concluded that preoperative anxiety was more common among young patients (16, 22, 26, 31, 41–43).

Larger proportion of previous studies on preoperative anxiety was done among elective surgical patients and considered preoperative anxiety as a prominent feature of elective surgery. Only few studies were published comparing preoperative anxiety between elective and emergency surgical patients. Preoperative anxiety was frequently observed among emergency surgical patients (44–46). In our study, the patients who came for emergency surgery were 2.5 times more likely to have preoperative anxiety than those who came for elective surgery (AOR: 2.5, CI: 1.3–4.7, *P*: 0.005). During emergency surgery, there is no enough time to adapt the situation and reassurance. Preoperative assessment and visits are known for anxiety-relieving effects, which are not feasible during emergency conditions (44). Previously, Woldegerima et al. have found that the prevalence of preoperative anxiety was 59.6% among elective surgical patients in the same hospital. In the current study, the prevalence of preoperative anxiety among elective surgical patients was minimized to 44.1%. This reduction might be explained by clinicians that might begin to give some attention to preoperative anxiety (22). In the current study, the prevalence of preoperative anxiety among emergency surgical patients was 67.8%. Kuzminskaitė et al. found that patients had preferred that conversations were the best options to reduce preoperative anxiety for which only limited time is available during emergency surgery (30).

Similarly, presence of moderate to severe pain during the preoperative period increased the occurrence of preoperative anxiety by nearly threefold (AOR: 2.6, CI: 1.2–5.4, *P*: 0.005). Consistently, Xu et al. stated that a higher VAS score during the preoperative period was associated with preoperative anxiety (32, 33). Among emergency surgical patients, most of the patients (71%) had suffered from moderate to severe pain, which, in turn, had significant association with preoperative anxiety.

Our current study revealed that the surgical patients who came from rural residencies were more anxious during the preoperative period (AOR: 1.8, CI: 1.1–2.9, *P*: 0.031). Charana et al. have justified this by additional difficulties patients were facing, such as longer distances to travel and the need of accommodation because of the surgery, the expenses, self-perception, diversified communication patterns, and lower tendency to obtain information (47). Despite the current finding, our previous study has shown that the patients who came from urban residencies were more anxious preoperatively (22, 41). A study done by Khalili et al. demonstrated comparable state anxiety between rural and urban residents, while urban residents had higher trait anxiety (41). The discrepancies might be explained by inclusion of emergency patients in the current study, which count for nearly two-third of the sample size (63.8%).

The patients who were scheduled to receive general anesthesia were found to have higher preoperative anxiety than those who were scheduled to receive regional anesthesia (69.7 vs. 51.9%). However, our data denied statistically significant associations between a plan of anesthesia and preoperative anxiety. Previously, studies have claimed that preoperative anxiety was predominant among patients who were waiting to be operated under general anesthesia, and they tended to require higher doses of anesthetic agents and poor recovery (5, 8, 19, 24, 31, 42, 48, 49). Potentially, this might be due to being awake and conscious of the surroundings, and no need of airway instrumentation under regional anesthesia. Whereas, under general anesthesia, the patients might think that they will not have control and will be at the goodwill of the healthcare professionals (24).

The factor commonly found associated with preoperative anxiety in many previous studies was sex. Female patients were more likely to experience preoperative anxiety than male patients, which was explained by hormonal differences, unique family bondage, higher trait anxiety, and emotional sensitivity of females (5, 19, 24–26, 31, 33, 42, 43, 50–52). Our results did not claim similarly as preoperative anxiety was frequently observed among the male patients than the female patients (66.1 vs. 54%) despite the absence of statistically significant association. Over half of the female patients were obstetric patients, and lesser anxiety was observed among the obstetric patients. This might be the possible cause for the discrepancy. Frot et al. have revealed higher anxiety among males than females (53), and other studies declared no statistically significant association between sex and preoperative anxiety (22, 54).

Inclusion of emergency surgical patients was the strength of the study. However, due to its cross-sectional design, it could not show temporal relationships among variables.

## Conclusion

The prevalence of preoperative anxiety among surgical patients was high. Being elderly (age ≥ 60 years), emergency surgery, preoperative pain, and rural residency were found significantly associated with preoperative anxiety. Assessment for preoperative anxiety should be a routine component of preoperative assessment of both elective and emergency surgical patients. Preoperative pain should be appropriately managed as it can help to reduce preoperative anxiety. Optimal anxiety reduction methods should be investigated and implemented in the hospital.

## Data availability statement

The raw data supporting the conclusions of this article will be made available by the authors, without undue reservation.

## Ethics statement

The studies involving human participants were reviewed and approved by the Ethical Review Committee of Department of Anesthesia, University of Gondar. The patients/participants provided their written informed consent to participate in this study.

## Author contributions

YW conceptualized the study objectives and developed the proposal. TB, GF, and MG criticized the proposal. YW and WC led the manuscript preparation. All authors participated in the data management and statistical analyses, read, and approved the final manuscript.

## Abbreviations

AOR: Adjusted Odds Ratio
ASA: American Society of Anesthesiology
CI: Confidence Interval
COR: Crude Odds Ratio
IBM: International Business Machines
IQR: Inter-quartile Range
SPSS: Statistical Package for the Social Sciences
STAI: State-Trait Anxiety Inventory
VAS: Visual Analog Scale
UoGCSH: University of Gondar Comprehensive Specialized Hospital.
